# Biocompatibility and Physico-Chemical Properties of Highly Porous PLA/HA Scaffolds for Bone Reconstruction

**DOI:** 10.3390/polym12122938

**Published:** 2020-12-09

**Authors:** Anna Zimina, Fedor Senatov, Rajan Choudhary, Evgeniy Kolesnikov, Natalya Anisimova, Mikhail Kiselevskiy, Polina Orlova, Natalia Strukova, Mariya Generalova, Vasily Manskikh, Alexander Gromov, Anna Karyagina

**Affiliations:** 1 Center for Composite Materials, National University of Science and Technology “MISIS”, Leninskiy Pr. 4, 119049 Moscow, Russia; senatov@misis.ru (F.S.); rajandeshwal@gmail.com (R.C.); kea.misis@gmail.com (E.K.); n_anisimova@list.ru (N.A.); kisele@inbox.ru (M.K.); 2 N. F. Gamaleya National Research Center of Epidemiology and Microbiology, Ministry of Health of the Russian Federation, Gamaleya Str. 18, 123098 Moscow, Russia; p.orlova88@gmail.com (P.O.); natalka.fanr@gmail.com (N.S.); keysi1986@mail.ru (M.G.); manskikh@mail.ru (V.M.); alexander.v.gromov@gmail.com (A.G.); akaryagina@gmail.com (A.K.); 3 N. N. Blokhin National Medical Research Centre of oncology of the Health Ministry of Russia, Kashirskoye sh. 24, 115478 Moscow, Russia; 4 A. N. Belozersky Institute of Physical and Chemical Biology, Lomonosov Moscow State University, 119992 Moscow, Russia; 5 All-Russia Research Institute of Agricultural Biotechnology, Timiryazevskaya Str. 42, 127550 Moscow, Russia

**Keywords:** polylactide, composites, surface wettability, porosity, cell viability, in vivo studies, maxillofacial reconstruction

## Abstract

The major problem in bone tissue engineering is the development of scaffolds which can simultaneously meet the requirements of porous structure, as well as have the ability to guide the regeneration of damaged tissue by biological fixation. Composites containing biodegradable matrix and bioactive filler are the new hope in this research field. Herein we employed a simple and facile solvent casting particulate-leaching method for producing polylactide acid/hydroxyapatite (PLA/HA) composites at room temperature. FT-IR analysis confirmed the existence of necessary functional groups associated with the PLA/HA composite, whereas energy-dispersive X-ray (EDX) spectra indicated the uniform distribution of hydroxyapatite particles in the polymer matrix. The beehive-like surface morphology of the composites revealed the presence of macropores, ranged from 300 to 400 μm, whereas the thickness of the pores was noticed to be 1–2 μm. The total porosity of the scaffolds, calculated by hydrostatic weighing, was found to be 79%. The water contact angle of pure PLA was decreased from 83.6 ± 1.91° to 62.4 ± 4.17° due to the addition of hydroxyapatite in the polymer matrix. Thus, the wettability of the polymeric biomaterial could be increased by preparing their composites with hydroxyapatite. The adhesion of multipotent mesenchymal stromal cells over the surface of PLA/HA scaffolds was 3.2 times (*p* = 0.03) higher than the pure PLA sample. Subcutaneous implantation in mice demonstrated a good tolerance of all tested porous scaffolds and widespread ingrowth of tissue into the implant pores. HA-containing scaffolds showed a less pronounced inflammatory response after two weeks of implantation compared to pure PLA. These observations suggest that PLA/HA composites have enormous potential for hard tissue engineering and restoring maxillofacial defects.

## 1. Introduction

Large scale bone imperfections in the craniomaxillofacial area occur during sports activities, traffic accidents, aging, and cancer, while progressive resorption of the alveolar bone after a dental loss can cause impairment of their structures, contributing to detectable dysfunction, as well as disfiguration [1]. Other important and prevalent causes of bone loss include periodontitis and periapical pathology [2]. The growth of bacteria at the periodontal ligaments damages the underlying jawbone leading to periodontal bone loss, whereas periapical pathology is associated with the occurrence of defects surrounding the tip of the root of a tooth. These traumatic defects of the facial area also have several adverse consequences that complicate the life of the affected person [3]. In severe cases, patients require surgery, including the replacement of damaged structures with a bone implant. Bone grafts have been utilized for over a decade to restore the normal functioning of the body and enhance human survivability. The limited supply, variability in the rate of resorption, and risk of infection during incision are the limitations associated with autografts. An allograft is obtained either from living donors or cadavers, and can have a demineralized bone matrix (unhealthy donors), or donor mismatch with the recipient, while xenografts face pathogenic transmission issues and immune rejection by the patient body [4,5]. These drawbacks restrict their applications in therapy.

Despite remarkable advancements in medical technology, the complete restoration of the complex 3D architecture of the craniomaxillofacial bone is another limitation. The good mechanical stability and fracture resistance of metallic biomaterials provide long-term implant performance reliability in load-bearing applications [6]. The conventional titanium plates are considered standard in implantology and maxillofacial surgery due to their biocompatibility [5]. However, the primary objective of the craniomaxillofacial bone restoration not only demands mechanical stability, but also requires simultaneously mimicking the morphological structure, as well as the recovery of normal functioning. Fulfilling these requirements is a healthcare problem and restoration of maxillofacial and oral defects is still a challenge in the biomedical field.

One of the most important tasks of modern maxillofacial surgery is to find an optimal implantable biomaterial that could enhance the reconstruction of the damaged tissue. The utilization of a bioresorbable scaffold could potentially address these limitations. Bioresorbable materials are safe, effective, and sufficiently flexible for maxillofacial surgery [7,8,9,10,11]. Therefore, such materials are more suitable than metallic or bioinert implants, which are used on a large scale in implantology. Polymers and copolymers of PLLA, poly-d-lactic acid (PDLA), polyglycolic acid (PGA), and polydioxanone sulfate are the most commonly explored [12]. Nevertheless, some problems associated with bioresorbable materials are poor mechanical strength, slow resorption time, and insufficient bioactivity [7,8,9].

The primary requirement for bone regeneration is that the scaffold should have the potential to mimic the microstructure of natural host tissues, and provide the necessary environment to promote cell proliferation and attachment of osteoblast cells. These properties can be achieved by employing the concept of tissue inspired biocomposites [13]. Natural bone is the best-known example of composite material in which apatite particles are embedded in collagen fibers. The flexibility of biocompatible polymers, combined with the bioactivity of ceramics in specific volume fractions, helps in the development of composites with improved functionalities [14]. Besides, the introduction of bioactive inorganic fillers into polymers makes it possible to achieve the desired kinetics of degradation and resorption of the polymer matrix. The use of scaffolds containing calcium phosphates as fillers has been widely explored for hard tissue regeneration. These inorganic components dissolve in the physiological environment, and stimulate bone formation [15]. The survival rate of such biomaterials has been found to be much higher compared to bioinert and metallic implants. The ceramic particles should not only be embedded inside the polymer matrix, but also cover the surface to improve the osteoconductivity of the composite material. Thus, the composite architecture (orientation, distribution, and percentage of reinforcements) and bonding between the reinforcement–matrix also play a key role. Effective control over these factors can assist in tailoring the mechanical and biological activity of the composites to meet the requirements of various biomedical applications [16]. Scientists across the globe are combining cells with natural as well as synthetic polymers (chitosan, collagen, polylactide, polycaprolactone, polyethylene glycol) and hydroxyapatite as fillers for the development of viable scaffolds for hard tissue regeneration applications [17,18,19,20]. Similarly, hydroxyapatite (HA) reinforced in polylactic acid (PLA) is considered one of the promising composite materials, and preferred in bone healing [21].

Earlier PLA/HA composites, with interconnected macroporous (200–600 µm) architecture and elastic modulus of 1.5 MPa to 0.15 MPa, were prepared for tissue engineering applications [22]. Si et al., (2019) fabricated PLA and PLA/HA scaffolds having more than 85% porosity [23]. A decline in the porosity of the samples and the hydrophilicity, mechanical stability, and cytocompatibility was enhanced as the concentration of hydroxyapatite was increased. Recently Backes et al., (2020) studied the interaction of PLA/HA composites with MG63 cells and found an increase in their proliferation rate, as compared to pure PLA [24]. PLA/PCL/HA composites prepared by 3D printing have shown macroporous structures (200–300 μm) with 77% porosity, and a Young’s modulus of 1.35 MPa [25]. Similarly, PLA/nano HA composites developed by additive manufacturing showed a compressive strength (23.36 ± 0.48 MPa) similar to that of cancellous bone [24]. These scaffolds promoted osteogenesis when implanted in the distal femora defect of a rabbit model. Wu et al., (2020) attempted the preparation of PLA/HA composites to mimic the structure of trabecular bone [25]. The mechanical behavior was similar to that of human bone, and the elastic modulus, as well as pullout load ability, was enhanced as the content of HA was increased in the PLA matrix. However, no additional biological properties were evaluated in this report.

Despite these promising in vitro studies published in the literature, a detailed investigation of the in vivo behavior of PLA/HA scaffolds is less explored. The current work aims to fill this gap, and attempts to carry out in vivo studies in addition to in vitro investigations to predict real-life applications in the biomedical field. Moreover, very few articles have been reported exploring the cytocompatibility of poly (lactic acid)/hydroxyapatite composites for the reconstruction of maxillofacial defects [26,27,28]. The present work deals with the preparation of porous PLA/HA scaffolds, and examines controlling their pore size by using solvent casting and a particulate leaching method. Hydroxyapatite nanoparticles were reinforced in different compositional ratios into the poly (lactic acid) matrix, and their influence on structure, morphology, wettability, and mechanical stability was studied. In addition, the biocompatibility of the scaffolds was evaluated by cell adhesion, cell proliferation, colorimetric assay using 3-(4, 5-dimethylthiazol-2-yl)-2, 5- diphenyltetrazolium bromide (MTT assay), and subcutaneous implantation in mice. The results obtained indicate that the porous PLA/HA scaffolds elaborated in this work have high biocompatibility, provide high efficiency of tissue ingrowth, and thus can be used for intraosseous implantation in reconstructive surgery.

## 2. Materials and Methods

### 2.1. Materials of Scaffolds

Dichloromethane (Manufacturer: LLC “PKF Galreahim”, Moscow, Russia) was used as a solvent to dissolve the polylactide. Polylactide (molecular weight of 110 kg/mol, Ingeo 4032D by Natureworks LLC) in pellet form was used as the main material for the preparation of the scaffold. Hydroxyapatite (HA needle particles 90 nm in size, Ca/P ratio of 1.67, “Polistom”, Moscow) was used as a component to increase the bioactivity of the product. Food-grade sodium chloride (NaCl) in granulometric form (up to 0.8 mm), having a mass fraction of not less than 99.7%, was used as a pore-forming agent, and was purchased from LLC “TDS”, Podolsk, Moscow region, Russia.

### 2.2. Scaffold Preparation Technique

The solvent casting and salt leaching methods were utilized for the preparation of polylactide/HA scaffolds of different concentrations: 0, 15, 20% wt. HA. The hydroxyapatite was ground to a fine powder using an agate mortar and pestle. Then, 1 gm of polylactide was dissolved in 15 mL dichloromethane. The beaker was covered with a watch glass and stirred at room temperature until a homogeneous solution of polylactide was obtained. To achieve a porosity of nearly 70% volume, 4 g of sodium chloride was added to the polylactide solution followed by a specified amount of hydroxyapatite powder. The resultant mixture was stirred constantly for 20 min, leading to the uniform dispersion of hydroxyapatite powders. The slurry mixture obtained was cast in a cylindrical mold (diameter 3.5 cm, height 0.4 cm), and the solvent was evaporated by drying at room temperature overnight. The scaffolds were removed from the mold and washed with double distilled water to induce porosity, with the elimination of dissolved salt. Finally, the composites were dried in a vacuum furnace to remove the dichloromethane vapors trapped inside the voids. The composites were washed with ethanol and stored in a desiccator until further use. A similar procedure was followed for the preparation of a pure polylactide scaffold without hydroxyapatite, in order to understand the variations in the properties of the composite material. The scaffolds for in vitro experiments were sterilized by autoclaving at 0.5 atm, 112 °C for 40 min.

### 2.3. Characterization of Scaffold

#### 2.3.1. Structure Characterization

The microstructure and elemental analysis of the scaffolds were studied by scanning electron microscopy (SEM) using a VEGA 3 TESCAN microscope coupled with energy-dispersive X-ray spectroscopy (EDX) (Oxford Instruments, Abingdon, UK). The presence of different functional groups present in the scaffold was analyzed by FT-IR spectroscopy (Nicolet 380 IR spectrometer, Thermo Scientific, Waltham, MA, USA) in the range of wavenumbers from 600 to 4000 cm^−1^.

#### 2.3.2. Study of Mechanical Properties

Mechanical tensile tests was performed on a universal testing machine Zwick/Roell Z 020, (Zwick GmbH & Co. KG, Ulm, Germany) using porous PLA, PLA/HA 15%, and PLA/HA 20% samples (8 samples for each composition, n = 8) in accordance with ISO 1926:2009. Samples of 1.7 mm thickness, with a working zone of 30 mm, were stretched at a speed of 2 mm / min without preloading. Additionally a force transducer, Zwick/Roell XForce P (Zwick GmbH & Co. KG, Ulm, Germany), and a longitudinal deformation transducer were used in the measurements.

#### 2.3.3. Measurement of the Contact Angle

The water contact angle was measured to analyze the hydrophilicity of the scaffolds by the sessile drop method, using EasyDrop wetting angle measurement equipment. Nearly 5 μL of distilled water was dropped on the surface of non-porous plane-parallel samples by the dosing system (ASTM D724-99 (2003)), and the contact angle was recorded after 2 s. Drop shape analysis software was used to determine the baseline and calculation of the contact angle. The test was repeated for each scaffold (n = 5), and the results are shown as means with standard deviation.

### 2.4. In Vitro Studies

#### 2.4.1. Cell Culture

The scaffolds (pure PLA, PLA/HA 15%, PLA/HA 20%) were molded into disks having 6 mm diameter and 3 mm height for cellular studies. All the scaffolds were initially sterilized in PBS (2 mL for each sample) and later placed carefully in a 24-well plate (Costar). Multipotential mesenchymal stromal cells (MMSC) were generated from feline bone marrow. The bone marrow was surgically obtained from a specific pathogen-free cat undergoing routine surgery. This procedure was conducted with the approval of the Local Ethical Committee of N.N. Blokhin NMRC of oncology, Moscow, Russia. The owner signed informed consent. The bone marrow cells were then made into a single cell suspension and plated into a T175 flask (Corning). The cells were cultured in RPMI-1640 medium (Thermo Fisher Scientific, Waltham, MD, USA) supplemented with 10% fetal bovine serum (FBS; HyClone Inc., Thermo Fisher Scientific, Waltham, MD, USA), 1% L-glutamine, and 1% penicillin/streptomycin (both from PanEco, Moscow, Russia) at 37 °C in 5% carbon dioxide. After 1 day, non-adherent cells were removed by washing twice with phosphate-buffered saline (PBS). MMSCs from passage 3 were used for experimentation.

#### 2.4.2. Cell Adhesion and Proliferation Analysis

Cells were trypsinized and washed two times in PBS, and then twice with RPMI-1640 medium. In the next stage the cells (930,000 cells/ 40 µL suspension) were seeded on the scaffolds (n = 9 of each type), or well bottom in controls, and incubated at 37 °C in 5% carbon dioxide for 15 min. Then, 0.96 mL of growth medium was carefully added to all the wells and incubated under similar conditions. Cells in control wells were intact. PLA, PLA/HA 15%, and PLA/HA 20% scaffolds (n = 9 of each type) were incubated with cells for 6 h. Non-adherent cells were removed by gently washing with PBS. Cell-seeded scaffolds were put in an empty plate well. Three scaffolds of each type were used for cell adhesion evaluation, and six scaffolds of each type were used for proliferation analysis. A part of the cell-seeded scaffolds (n = 6 of each type) were cultured in a fresh portion of growth medium at 37 °C in 5% carbon dioxide for 2 days.

The cell adhesion ability of the samples was investigated by using a Pierce lactate dehydrogenase (LDH) Cytotoxicity Assay Kit (Thermo Fisher Scientific, Waltham, MD, USA). The cells were adhered to the disk-shaped samples and lysed with 0.5% Triton X-100 solution in PBS. Similarly, the procedure was followed for the control samples. The release of LDH was measured as per the manufacturer’s instructions: Experimental LDH release in wells with three tested scaffolds, and maximum LDH release in the control wells. The formazan concentration was determined by measuring optical density (OD) in triplicate at 492 nm in a 96-well plate (Costar) with a plate reader (MS Multiscan, Labsystem, Thermo Fisher Scientific, Waltham, MD, USA). RPMI-1640 medium was used for this assay to eliminate optical interference between phenol red and the red formazan product. The average values of the culture medium background were subtracted from all values of the experimental and control wells. Equation (1) was used to compute the percentage of cell adhesion.

(1) $$\mathrm{Cell}\;\mathrm{adhesion}\;\left(\%\right)=\frac{\mathrm{Experimental}\;\mathrm{LDH}\;\mathrm{Release}\;\left(\mathrm{OD}\;492\right)}{\mathrm{Maximum}\;\mathrm{LDH}\;\mathrm{Release}\;\left(\mathrm{OD}\;492\right)}\times 100$$

Cell proliferation was investigated by measuring the release of LDH after 48 h incubation, in contrast to the release of LDH after the first 6 h of incubation. Briefly, PLA, PLA/HA 15%, and PLA/HA 20% scaffolds (n = 3 of each type) with adherent cells were removed from growth medium after 2 days of incubation and gently washed with PBS. Later, the experimental LDH release was measured after 24 h, as described above. Experimental LDH release after 6 h was the average value of Experimental LDH release computed for cell adhesion analysis. The cell proliferation on the surface of the scaffolds was calculated according to Equation (2).

(2) $$\mathrm{Cell}\;\mathrm{proliferation}\;\left(\%\right)=\frac{\mathrm{Experimental}\;\mathrm{LDH}\;\mathrm{Release}\;\mathrm{after}\;48\;h\;\left(\mathrm{OD}\;492\right)}{\mathrm{Experimental}\;\mathrm{LDH}\;\mathrm{Release}\;\mathrm{after}\;6\;h\;\left(\mathrm{OD}\;492\right)}\times 100-100$$

An MTT assay was employed to study the survival of cells when placed in contact with the samples. The procedure followed is described elsewhere [29]. Briefly, MTT (3-(4,5-dimethylthiazol-2-yl)-2,5-diphenyltetrazolium bromide) of 50 µl volume was added into the growth medium in wells with cell-seeded scaffolds, and incubated for 4 h at 37 °C in 5% carbon dioxide. Later the growth medium was replaced with 500 µL dimethyl sulfoxide in each well-containing scaffolds. The formazan concentration was determined by measuring OD at 540 nm in a 96-well plate with a plate reader (MS Multiscan, Labsystem, Thermo Fisher Scientific, Waltham, MD, USA). The experiments were performed in triplicate, and the relative cell viability was expressed relative to the control cells. The morphology and number of adhered cells were investigated with an Axioplan-2 Imaging Light/ Fluorescent Microscope (Carl Zeiss). Cells were stained by hematoxylin and eosin.

### 2.5. In Vivo Implantation and Histologic Analysis

Subcutaneous implantation was performed on nine males of outbred ICR (CD-1) mice aged 38–47 days. The study was approved by the Committee on Biomedical Ethics of the N.F. Gamaleya National Research Center for Epidemiology and Microbiology (Protocol No. 17). All procedures with animals were conducted according to the Guide for the Care and Use of Laboratory Animals. The animals were divided into 3 groups (3 animals per group). Disks (diameter 4 ± 0.1 mm, thickness 1 ± 0.1 mm) from pure PLA were implanted in group 1, disks from PLA/HA 15% in group 2, and disks from PLA/HA 20% in group 3 (2 discs per animal). The animals were anesthetized by using intraperitoneal injection with a mixture of tiletamine and zolazepam (Zoletil 100, Virbac, Carros, France), 30 mg/kg, and Xylazine, 10 mg/kg. After shaving and disinfection of the rostral subscapular region, a linear skin incision of 1.0 cm was made. The sterile discs were surgically implanted into the subcutaneous pockets. Finally, the wound was sutured. After 14 days, the animals were euthanized by carbon dioxide inhalation. The samples, including surrounding tissues, were harvested from each mouse, immersed immediately in 4% buffered paraformaldehyde for 24 h at room temperature, dehydrated in an IsoPrep™ (Biovitrum, Saint-Petersburg, Russia) series, and embedded in paraffin. Later samples were sectioned at 5 µm, stained with hematoxylin and eosin (H&E), and analyzed using an AxioScope A1 Microscope, Carl Zeiss, Oberkochen, Germany.

A semi-quantitative scoring system was used to assess the histological local response parameters based on the international standards ISO 10993–6 (2007). The assessed parameters included: the extent of inflammation, based on the number and type of inflammatory cells present; the presence of necrosis; the presence and extent of fibrosis/fibrous capsule; and the presence of fatty infiltration. The average score was first evaluated for each sample in 5 high powered (400×) fields (hpfs) of view in accordance with the criteria adopted in ISO 10993-6. Then the Score for the group was determined. For the presence of neutrophils, lymphocytes, plasma cells, and macrophages: grade 0 corresponds to the absence of cells in the evaluated area (0 cells per hpf); grade 1-with minimal number of cells (1–5 cells/hpf); grade 2-mild (5–10/hpf); grade 3-moderate (>10/hpf); grade 4-severe, packed with cells. For the giant multinucleated cells: grade 0 corresponds to 0 cells per hpf; grade 1-minimal number of cells (1–2 cells/hpf); grade 2-mild (3–5/hpf); grade 3-moderate, heavy infiltrate (>10/hpf); grade 4-severe, sheets of cells. Necrosis: 0-absence of necrotic tissue; 1-minimal amount; 2-mild; 3-moderate; 4-severe. Fibrosis: 0-no fibrotic tissue; 1-minimal, narrow band of fibrotic tissue; 2-mild, moderately thick band; 3-moderate, thick band; 4-severe, extensive band. Fatty infiltrate: 0-no fatty infiltrate; 1-minimal, minimal amount of fat associated with fibrosis; 2-mild, several layers of fat and fibrosis; 3-moderate, elongated and broad accumulation of fat cells about the implant site; 4-severe, extensive fat completely surrounding the implant.

### 2.6. Statistics

All the data were expressed as means ± standard error of the mean. The activity of the scaffolds were compared with each other, and with controls, in the Mann–Whitney U-test. Differences were considered significant at *p* < 0.05.

## 3. Results and Discussions

### 3.1. Microstructure Analysis

**Figure 1:**
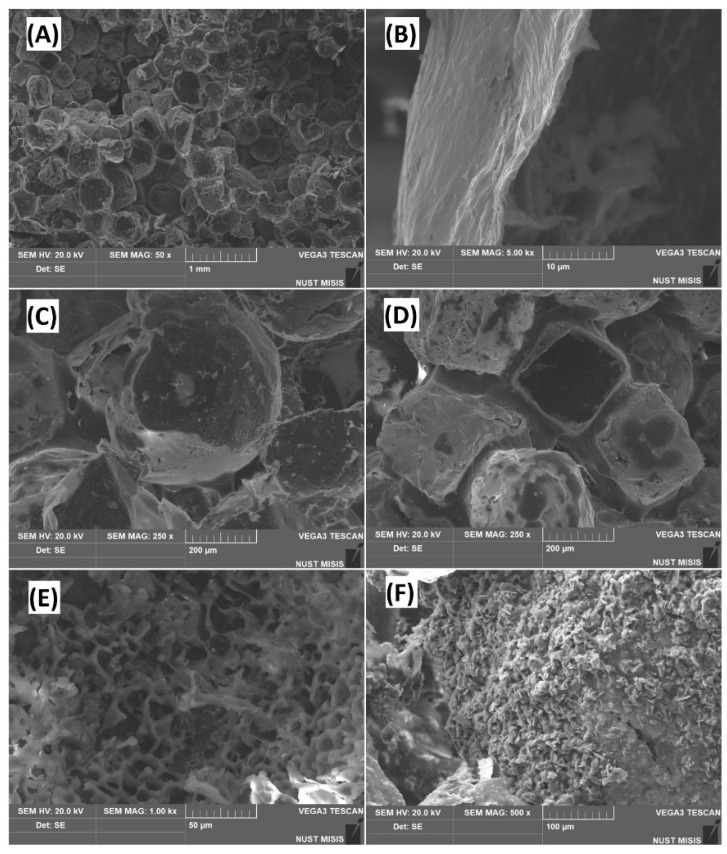
The microstructure of polylactide acid/hydroxyapatite (PLA/HA) composite sample: general view (**A**); pore “wall” (**B**); spherical (**C**), and cubic (**D**) pores; microrelief (**E**); aggregated hydroxyapatite (HA) particles (**F**).

Figure 1 shows that the surface of the scaffold was covered by a network of intact porous structures. The surface of the samples at the microscopic level appeared to possess a beehive-like morphology (Figure 1A). Uniform porosity was noted over the entire height of the sample. A micrograph of the full slice of sample is shown in Figure S1 in the Supplementary material. The macropores ranged from 300 to 400 μm, whereas the thickness of the pore walls was noticed to be 1–2 μm. This pore size was found to be favorable for stimulating the proliferation and differentiation of osteoblast cells [30,31]. The geometries of the pores varied from spherical to cubic (Figure 1C,D). This was because the shape of pores depends on the geometry of the template particles (NaCl). The interaction between spherical pores is better, and the probability for their fusion is greater compared to cubic pores. Zhang et al. suggested that scaffolds composed of spherical pores are mechanically more stable than those with cubic geometry [32]. The surface of the pure polylactide scaffold exhibited the appearance of microrelief, and the size of the protrusions was detected as 5–15 μm (Figure 1E). In the case of the PLA/HA composite (Figure 1F) the surface was composed of aggregated needle-shaped hydroxyapatite particles having a dimension of 10–20 μm.

### 3.2. Porosity Calculation

Scaffolds for tissue regeneration should possess a porous architecture. The porous structure assists in providing the necessary environment to promote cell migration, vascularization, tissue ingrowth, nutrient delivery to the regenerating tissue, and facilitates the removal of metabolic products. Amongst different criteria, porosity is considered as a key factor for assessing the pore architecture of scaffolds. Vadgama stated that porous scaffolds are conceived as valuable materials for biological applications [33]. The porosity of the prepared samples was studied by using ImageJ software, and the total percentage of porosity was 74% vol. The micrographs obtained after processing the images are shown in Figure S2 in the Supplementary material section.

Moreover, the porosity of the samples was confirmed by hydrostatic weighing, as per Equation (3). According to the calculation, the total porosity of the samples was found to be 79%. This observation indicated that the formation of additional pores in the sample might be due to the creation of air bubbles during the addition of hydroxyapatite and salt particles in the polymer mixture, or owing to evaporation of dichloromethane.

(3) $$P=\left(1-\frac{m}{V\cdot \rho }\right)\times 100\%$$

where *P* is the porosity of the samples in %; *m* is the mass of porous samples, g; *V* is the volume of porous samples, cm^3^; and *ρ* is the theoretical density, g/cm^3^.

### 3.3. Sample Composition Analysis

**Figure 2:**
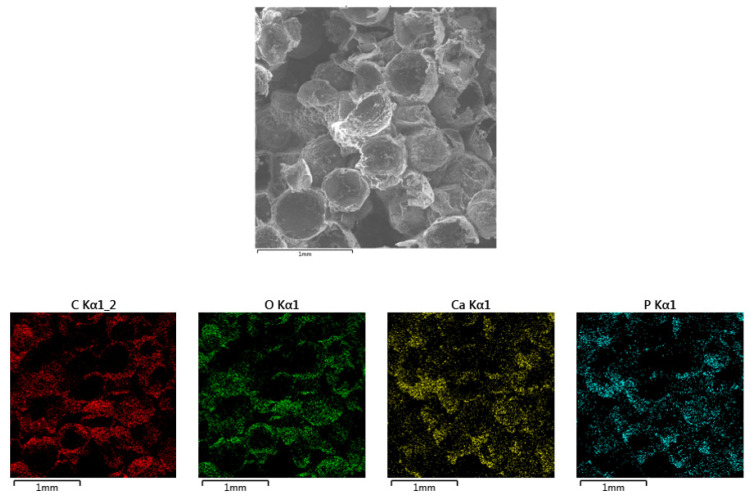
Elemental analysis and distribution of carbon, oxygen, calcium, and phosphorus in the sample.

The elemental composition and their distribution in the samples (PLA/HA 20%) were analyzed by energy-dispersive X-ray spectroscopy (Figure 2). The absence of impurities in the prepared samples showed the purity of the scaffolds. The uniform distribution of Ca, P, and O in the polylactide matrix indicated the fabrication of a homogeneous scaffold, and their presence confirmed the chemical composition of hydroxyapatite.

### 3.4. Mechanical Testing

An ideal biomaterial should provide sufficient structural support to the regenerating tissues during the remodeling or degradation process. In the present study, the mechanical behavior of the samples was also studied (Table 1). The Young’s modulus of pure PLA, PLA/HA 15%, and PLA/HA 20% were found to be 89.01 ± 12.74 MPa, 177.24 ± 53.62, and 183.56 ± 39.60 MPa, respectively. This observation indicated that the Young’s modulus of the samples was enhanced as the concentration of hydroxyapatite was increased in the polymer matrix. The Young’s modulus of the PLA/HA 15% and PLA/HA 20% scaffolds was two times greater than pure PLA. This is because the tensile modulus of HA is more than PLA [34]. However, the influence of the HA content on the tensile strength of samples was found to be negligible. The literature indicated that the mechanical properties of PLA/HA composites are highly dependent on the dispersion of HA in the PLA matrix [35]. Moreover, a sudden decrease in the value of elongation at breaking was noticed. Since polymers are flexible in nature, they possess a higher value of elongation at breaking, whereas the brittle nature of ceramics makes them have a lower value for elongation at breaking. Thus the flexibility of the scaffolds was reduced due to the incorporation of HA in the PLA matrix. A similar observation was made elsewhere [36]. Larranaga et al., (2014) reported similar findings for the measurement of tensile strength and elongation at break [37]. Due to the fact that the composite has a lower tensile strength value than bone, implants made of this material can be used in unloaded areas of the body, for example, in the maxillofacial area.

### 3.5. FT-IR Analysis

**Figure 3:**
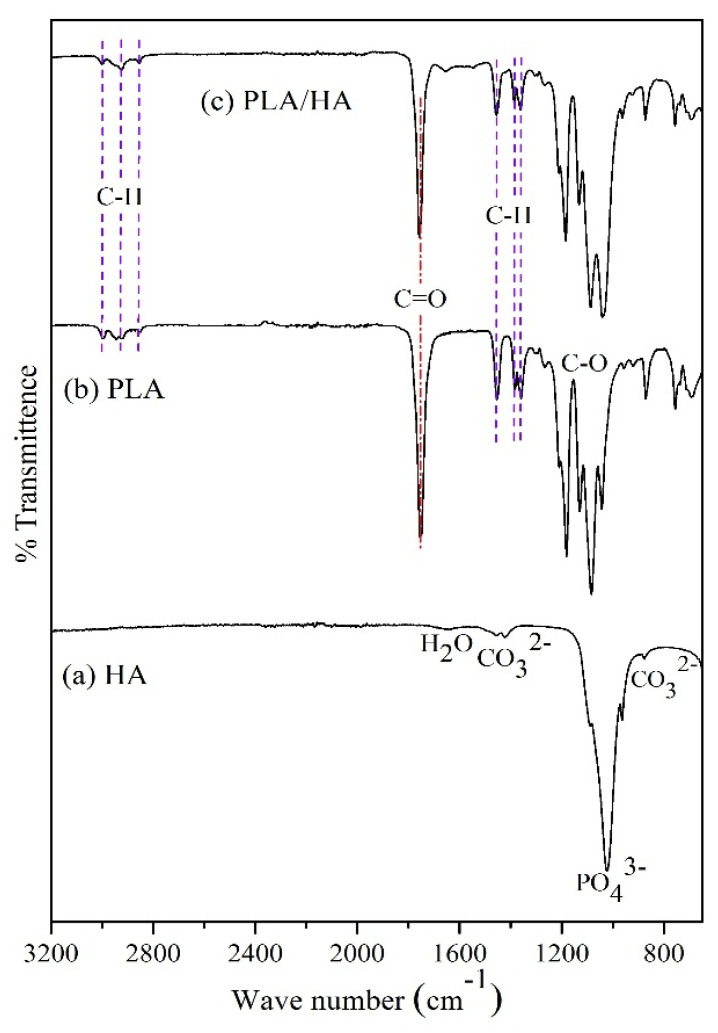
FT-IR spectrogram of (**a**) HA, (**b**) PLA, and (**c**) PLA/HA 15%.

The existence of different functional groups associated with hydroxyapatite, PLA, PLA/HA composites, and their comparison with each other was analyzed by FT-IR spectroscopy (Figure 3). The stretching vibration bands of a phosphate group (PO_4_^3−^) were detected at 964 cm^−1^, 1022 cm^−1^, and 1095 cm^−1^ respectively. The weak band around 1640 cm^−1^ corresponds to H_2_O absorption. The peaks at about 1419 cm^−1^ and 877 cm^−1^ were attributed to asymmetric stretching and bending vibrations of the carbonate group. This indicates the substitution of a carbonate ion at the phosphate ion site. These functional groups confirmed the presence of hydroxyapatite. The absorption bands related to pure PLA can be summarized as the bands at 2918 cm^−1^ and 2848 cm^−1^, which corresponds to asymmetric and symmetric C–H stretching of CH_3_. The asymmetric and symmetric bending modes of the methyl group were noticed at 1454 cm^−1^ to 1360 cm^−1^. The stretching vibrations of C=O (carbonyl group of the ester) were found at 1753 cm^−1^. The peaks ranging from 1043 cm^−1^ to 1180 cm^−1^ and 1083 cm^−1^ were attributed to C–O stretching in esters. Vibration of the C–COO bond was noticed at 871 cm^−1^, and the band at 755 cm^−1^ revealed the crystalline phase of polylactic acid [28,38,39].

The absorption spectra of PLA/HA composite (Figure 3c) were found to be similar to that of PLA (Figure 3b), and showing the vibrational bands associated with the polymer structure. This was either due to the overlapping of vibration bands of hydroxyapatite (850 cm^−1^–1100 cm^−1^) with the polymer, or because the occurrence of the phosphate group in the composite was much less in comparison to C–H bonds from the polymer chain. However, a minor shift in the intensity of the peak was observed. The intensity of the band around 1083 cm^−1^ got reduced, whereas the intensity of the band at 1043 cm^−1^ got enhanced (Figure 3c), as compared to the PLA spectra (Figure 3b). Thus, the appearance of a broadband in the regions from 1000 cm^−1^ to 1100 cm^−1^ was observed after preparing PLA/HA composites. This finding suggests the occurrence of some interference between the polymer and hydroxyapatite.

### 3.6. Assessment of Surface Hydrophilicity

**Figure 4:**
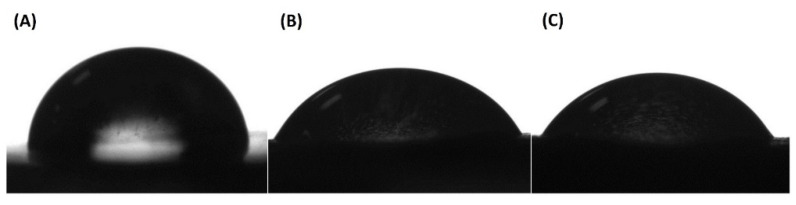
Image of droplets on the surface of samples of PLA (**A**), PLA/HA 15% (**B**), and PLA/HA 20% (**C**).

The surface hydrophilicity of a material is considered as a primary requirement to predict its interaction with cells and biological mediums. Different studies have suggested that wettability plays a key role in determining the potential of a bone scaffold to stimulate cell adhesion, proliferation, and bone osteointegration [40,41,42]. The hydrophobic nature of PLA limits its application in the biomedical field. Thus, aiming to enhance the hydrophilicity of PLA scaffolds, water contact angle studies were carried out. The results obtained in the current study indicated an improvement in the hydrophilicity of the composites. Table 2 shows the contact angle values of all the samples was less than 90°. Moreover, the contact angle was found to decrease with the increase in the hydroxyapatite content in the scaffolds. For instance, the surface of pure PLA had a water contact angle of 83.6 ± 1.91°, and with the addition of 15 and 20% hydroxyapatite in the PLA matrix, the contact angle dropped to 64.6 ± 1.87° and 62.4 ± 4.17°, respectively. This observation shows a decrease in the value of the contact angle of scaffolds by more than 20°. The potential reason for such an observation is that hydroxyapatite is a hydrophilic biomaterial, as it contains hydroxyl (OH) groups. Therefore, the incorporation of hydroxyapatite in the PLA matrix assisted in increasing the hydrophilicity of the composites. This observation can be correlated with the illustration shown in Figure 4, which indicated a constant decrease in the contact angle of the composites. This finding suggests that the surface hydrophobicity of a polymeric scaffold can be reduced by fabricating its composites with hydroxyapatite.

### 3.7. In Vitro Experiment

#### 3.7.1. Cell Adhesion

**Figure 5:**
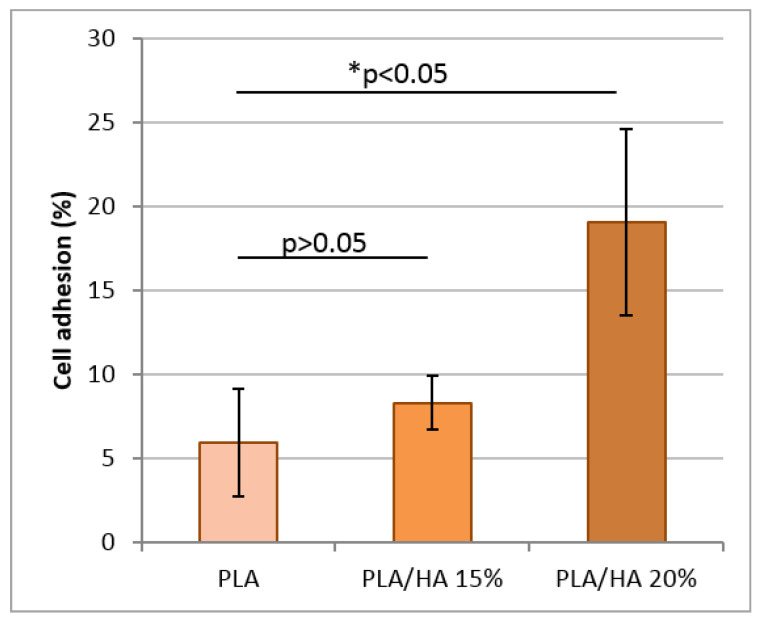
Percentage of multipotential mesenchymal stromal cell (MMSC) adhesion to PLA, PLA/HA 15%, and PLA/HA 20% versus control after incubation for 6 h at 37 °C in 5% carbon dioxide. * *p* < 0.05.

The main objective of the present work was to study the cytocompatibility of pure PLA and PLA/HA scaffolds to predict their biomedical applications. This was investigated by analyzing the adhesion of MMSC on the surface of scaffolds after 6 h of incubation (Figure 5). It was noticed that the composites containing fifteen and twenty percent bioactive hydroxyapatite revealed a cellular adhesion of nearly 8% and 19%, respectively. Whereas pure the PLA scaffold allowed only 6% of cells to adhere to its surface. This observation indicated that the activity of PLA/HA 20% was 3.2 times (*p* = 0.03) higher than the pure PLA sample. Literature reports have suggested that material composition, surface topography, surface chemistry (wettability), and microstructure (porosity, interconnectivity, pore shape, pore size) significantly influences cell adhesion on the surface of biomaterials [43,44]. Thus, the presence of these characteristics in PLA/HA composites resulted in an increased rate of cell adhesion over their surface as compared to pure PLA. Statistical analysis did not find significant differences between the activity of scaffolds with HA (*p* > 0.05).

#### 3.7.2. Cell Proliferation

**Figure 6:**
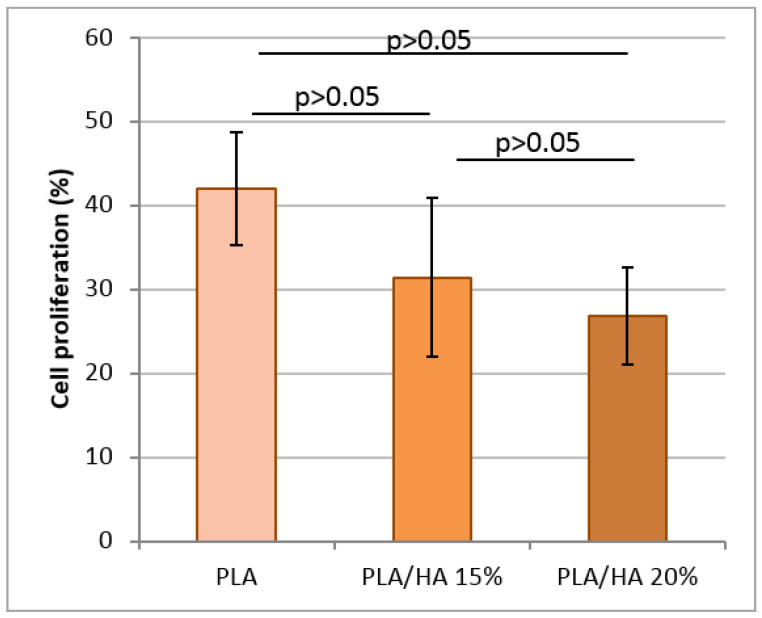
Percentage MMSC proliferation on PLA, PLA/HA 15%, and PLA/HA 20% scaffolds after cultivation for 2 days at 37 °C in 5% carbon dioxide.

All tested scaffolds provided MMSC proliferation, because after 2 days of incubation the amount of cell cytosolic LDH was higher by 27–42% than on the first day (Figure 6). It seems that the PLA scaffolds allowed cells to proliferate more intensively than the HA containing scaffolds (*p* > 0.05). In the present study, however, these results were not confirmed. No statistical differences were found between PLA/HA 15% and PLA/HA 20% scaffolds, or among the HA containing scaffolds in comparison with the PLA scaffolds, which were used as a control (*p* > 0.05).

#### 3.7.3. Cell Viability

**Figure 7:**
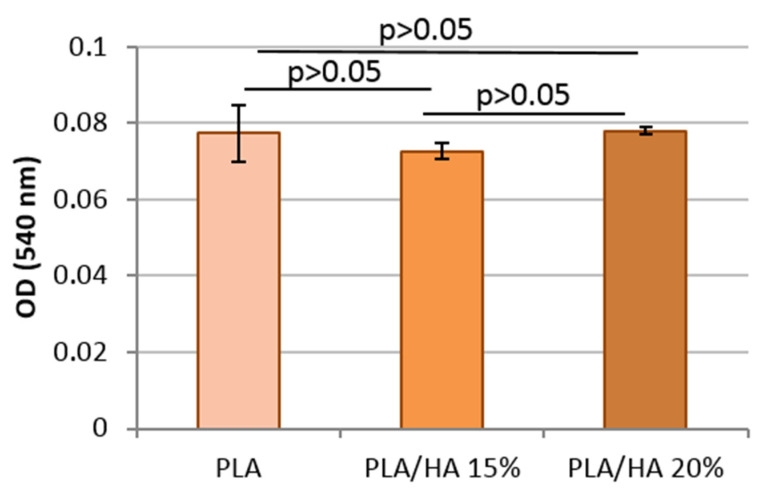
Formazan produced by MMSC seeded on PLA, PLA/HA 15%, and PLA/HA 20% scaffolds determined by MTT assay.

**Figure 8:**
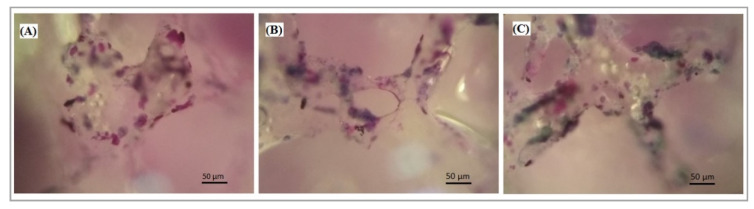
MMSC seeded on PLA (**A**), PLA/HA 15% (**B**), and PLA/HA 20% (**C**) scaffolds at 48 h of cultivation determined with light microscopy. Hematoxylin and eosin staining.

The viability of the MMSC cells cultured over the surface of the scaffolds was assessed by MTT assay. MTT assay is the most convenient test to study in vitro cellular behavior of materials. Figure 7 shows that the scaffold colonized with MMSC produced a large amount of formazan product. This revealed that the cells were able to metabolize MTT, which promoted the survival of the cells. Accordingly, the test showed that viable cells existed in scaffolds even after 48 h of incubation, making them quite suitable for the growth of MMSCs. Amongst all the samples, the highest cell viability was noticed for the PLA/HA 20% scaffold. Statistical analysis did not confirm the differences between the properties of the tested scaffolds (*p* > 0.05). The active colonization of cells on the surface of the scaffolds was analyzed by light microscopy. Figure 8 revealed a negligible difference in the cell number and morphology amongst the different scaffolds. Thus, pure PLA and PLA/HA scaffolds provided the necessary environment to support and guide the ingrowth of cells throughout the matrix of the composites.

These findings indicated that the scaffold containing PLA/HA possessed good biocompatibility, due to the presence of hydroxyapatite nanoparticles spread over the surface of the scaffolds. This might have enhanced the interaction between the bioactive hydroxyapatite and the cells, promoting the survival of the cells.

### 3.8. In Vivo Implantation and Histologic Analysis

**Figure 9:**
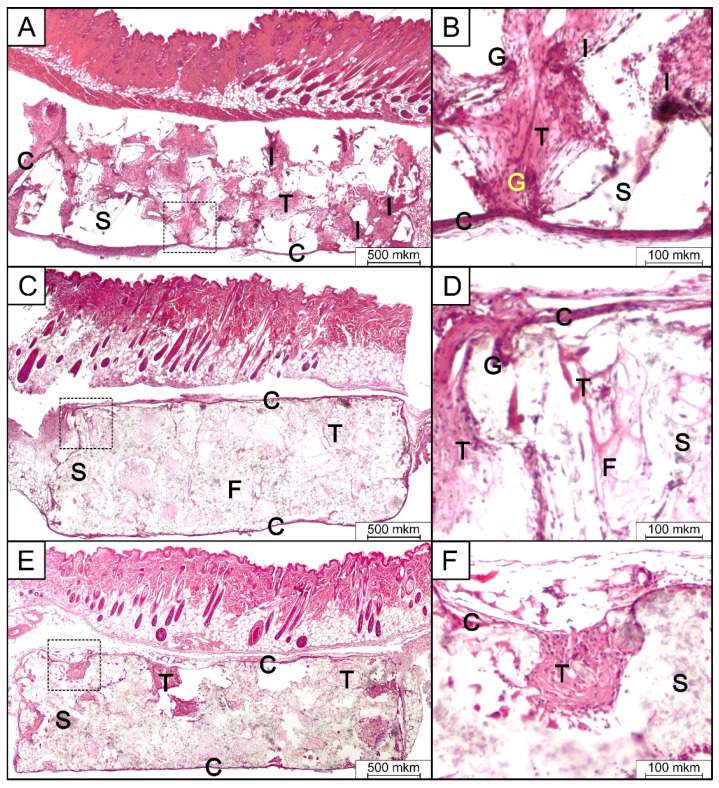
Light micrographs of H&E stained tissue sections of the subcutaneous implantation area in group 1 (PLA) (**A**,**B**), group 2 (PLA/HA 15%) (**C**,**D**), and group 3 (PLA/HA 20%) (**E**,**F**) after 2 weeks of implantation. S-scaffold; C-connective tissue capsule; T-connective tissue; F-collagen fibers; I-inflammatory infiltrates; G-giant multinucleated cell.

Histological analysis of pure PLA, PLA/HA 15%, and PLA/HA 20% samples implanted subcutaneously in mice for 2 weeks demonstrated very similar tissue reaction among all the cases (Figure 9). The epidermis and its appendages were found to be intact. In the subcutaneous tissue, the implant was located in the form of a porous disk, without any signs of resorption or perifocal osteogenesis. The presence of connective tissues in the pores, with different degrees of maturity and consisting of bundles of collagen of different sizes, was noticed. A number of giant multinucleated cells near and within the implanted device were also observed. Fibroblast-like cells and a moderate number of inflammatory effector cells (neutrophils, lymphocytes, and macrophages), which form focal infiltrates with a small number of thin-walled vessels, were also observed. On the periphery of the implant, there was a weak inflammatory reaction, with the formation of a connective tissue capsule of fibroblast-like cells and macrophages. There was also a poorly expressed infiltration of lymphocytes and plasmocytes. Pure PLA samples revealed the existence of a slightly more pronounced inflammatory reaction compared to the PLA/HA 15% and PLA/HA 20% scaffolds, as evidenced by the presence of more neutrophils, lymphocytes, and giant multinucleated cells (Table 3).

A weakly expressed inflammatory reaction, accompanied by the formation of a connective tissue capsule around the implant is a normal tissue reaction to a foreign body [45,46,47]. The absence of acute or chronic active inflammation, necrosis, and mineralization indicated good tolerance of the tested materials. A more pronounced inflammatory reaction in the case of pure PLA was probably a consequence of its greater hydrophobicity compared to the HA-containing scaffolds.

## 4. Conclusions

The current work reports the fabrication of porous PLA/HA composite for bone reconstruction applications. The composites were structurally (FT-IR), morphologically (SEM), elementally (EDX), and mechanically characterized. The solvent casting-assisted particulate leaching method resulted in the formation of composite having a network of intact porous structures. It was found that the shape of pores was dependent on the geometry of the porogen (NaCl) particles. The incorporation of hydroxyapatite in the PLA matrix not only enhanced the hydrophilicity of the composites, but also created a microrelief on the surface of the polylactide samples. This also changed the tensile properties of the polylactide. In this sense, the Young’s modulus of the PLA scaffolds increased with the addition of HA particles. In turn, the elongation at break and the ultimate strength of the scaffolds were reduced when the hydroxyapatite particles were included. Therefore, porous PLA/HA scaffolds may be suitable for soft tissue engineering applications, where materials displaying an elastomeric behavior are highly appreciated. The minor variations in the absorption bands of the composites revealed the existence of interference between the polymer chain and the hydroxyapatite. However, the appearance of no new functional groups was noticed in the PLA/HA scaffolds. The cellular studies expressed that the scaffolds had the potential to stimulate cell adhesion and growth. A good tolerance of all scaffolds, with minimal inflammatory response in the case of the HA-containing samples, was demonstrated as a result of in vivo implantation. A weak inflammatory response, and good tissue ingrowth into the pores of the scaffolds, 2 weeks after implantation allows us to hope for the successful use of porous PLA/HA composites for intraosseous implantation, including combined usage with bone growth inductor proteins, such as BMP-2.

## Figures and Tables

**Table 1:** Values of ultimate strength, Young’s modulus, and elongation at break obtained from mechanical tensile tests for PLA, PLA/HA 15%, and PLA/HA 20%.

Samples	Ultimate Strength, MPa	Young’s Modulus, MPa	Elongation at Break, %
Cancellous Bone	80–90	50–500	<1
PLA	1.53 ± 0.22	89.01 ± 12.74	6.42 ± 2.0
PLA/HA 15%	2.47 ± 0.19	177.24 ± 53.62	2.23 ± 0.54
PLA/HA 20%	1.41 ± 0.37	183.56 ± 39.60	1.15 ± 0.55

**Table 2:** Values of contact wetting angles of sample’s surfaces.

Sample	The Value of the Wetting Angle, °
PLA	83.6 ± 1.91
PLA/HA 15%	64.6 ± 1.87
PLA/HA 20%	62.4 ± 4.17

**Table 3:** Semi-quantitative evaluation of the histological local response parameters 2 weeks after subcutaneous implantation of PLA, PLA/HA 15%, and PLA/HA 20% samples.

	PLA	PLA/HA 15%	PLA/HA 20%
Polymorphonuclear cells	2 *	0	1
Lymphocytes	3	2	1
Plasma cells	1	1	1
Macrophages	1	1	1
Giant cells	2	1	1
Necrosis	0	0	0
Fibrosis	1	1	1
Fatty infiltrate	0	0	0

* Mean severity score (see Material and Methods, Section 2.5).

## Data Availability

The raw/processed data required to reproduce these findings cannot be shared at this time due to technical or time limitations.

## Supplementary Materials

The following are available online at https://www.mdpi.com/2073-4360/12/12/2938/s1, Figure S1: SEM image of the full slice of PLA/HA porous sample, Figure S2: Image of microstructure before (A) and after (B) processing in Image J.
