# Supplementary material for: Biocompatibility and Physico-Chemical Properties of Highly Porous PLA/HA Scaffolds for Bone Reconstruction
Source: Polymers (Basel). 2020 Dec 9;12(12):2938. doi: 10.3390/polym12122938 (PMC7764020; doi:10.3390/polym12122938)
Supplement: Supplementary file 1 [file polymers-12-02938-s001.pdf]

Supplementary Material:

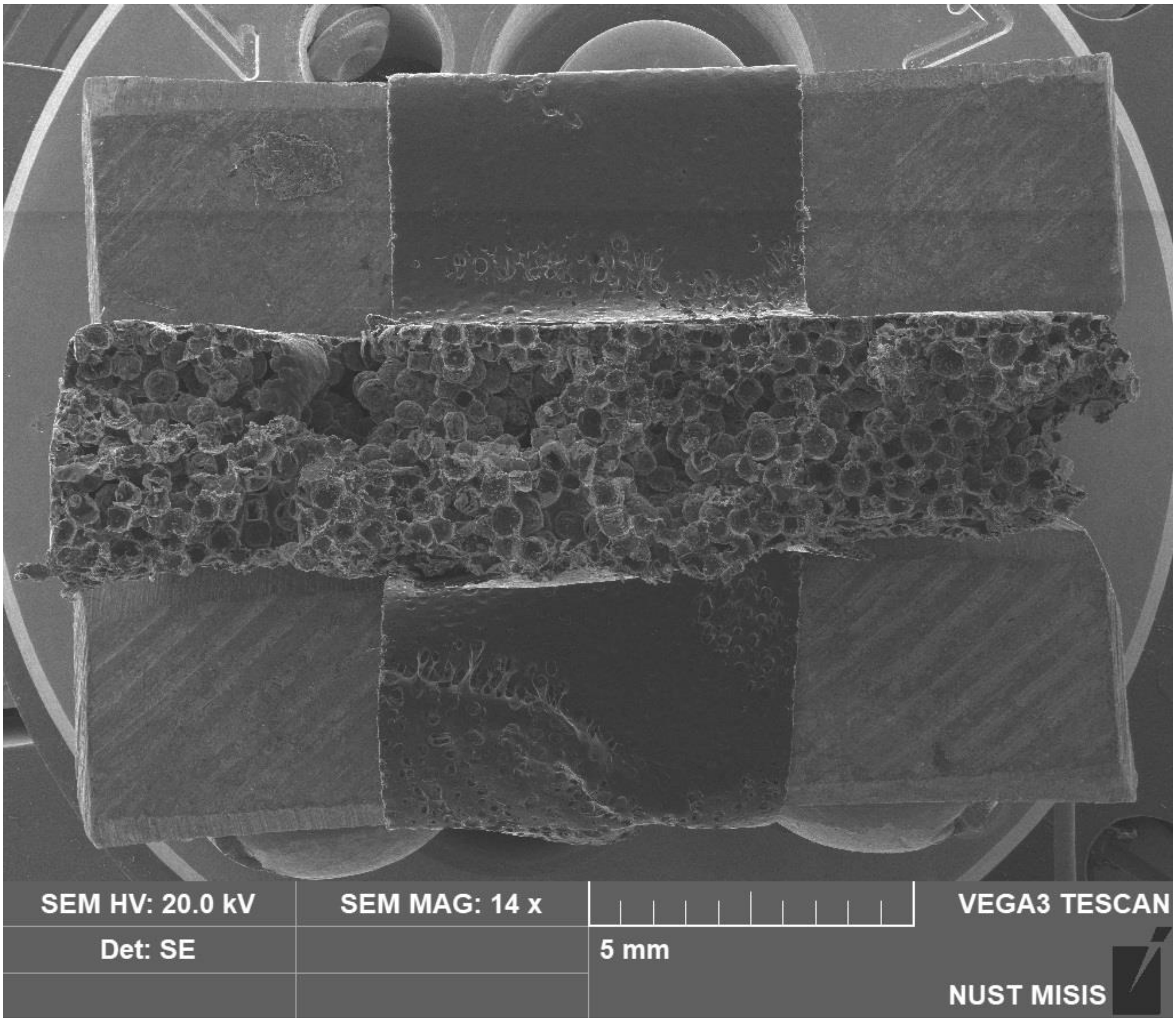

Figure S1 – SEM image of the full slice of PLA/HA porous sample

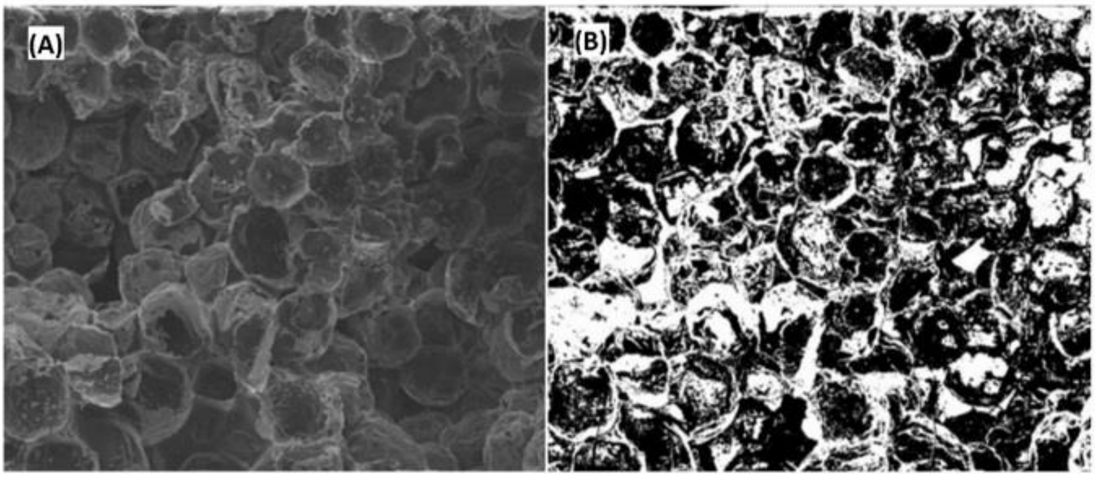

Figure S2 – Image of microstructure before (A) and after (B) processing in Image J
